# Depression and anxiety are associated with increased complications after penile prosthesis surgery: a retrospective cohort study

**DOI:** 10.1038/s41443-025-01149-9

**Published:** 2025-08-06

**Authors:** Mark Xu, Connor Policastro, Dylan Wolff, Mary Namugosa, Rory Ritts, Megan Escott, Ryan Terlecki

**Affiliations:** Department of Urology, Wake Forest Baptist Medical Center, Winston-Salem, NC USA

**Keywords:** Erectile dysfunction, Risk factors, Surgery, Adverse effects

## Abstract

Depression and anxiety are often comorbid with erectile dysfunction and are linked to worse surgical outcomes. We aimed to determine if depression/anxiety increased complication rates in inflatable penile prosthesis (IPP) surgery. All IPP cases by a single surgeon at our institution from 2020–2022 were reviewed. Data was collected on demographics, medical/psychiatric history, intraoperative details, and post-operative outcomes. Univariate, multivariate, and survival analysis were performed to assess relationships between patient factors and complications. 279 IPP cases were performed. Anxiety/depression was significantly associated with post operative complications (*p* = 0.002) and infection (*p* = 0.024). Anxiety/depression is independently associated with increased complications in a multivariable logistic regression model including age, BMI, diabetes, primary surgery, smoking status, pelvic surgery, radiation, Peyronie’s disease, and correctly holding anticoagulation. Survival analysis showed that anxiety and depression both resulted in faster times to complication and re-operation (all *p* < 0.05). This is the first study assessing the impact of mental health on IPP outcomes. Urologists should consider mental health when evaluating patients for IPP surgery, and mental health treatment prior to surgery may improve outcomes.

## Introduction

Impaired wound healing underlies several proposed risk factors for tissue and/or device infection after penile prosthesis surgery, such as diabetes, steroid use and spinal cord injury [1]. The overall rate of infection after penile prosthesis placement is roughly 2–3% across implant surgeries, though lower rates are reported after the advent of antibiotic coating/washouts [2–5]. Infections account for approximately half of complications in the immediate postoperative period per the national surgical quality improvement program. Prosthetic infections can be challenging to eradicate, potentially due to biofilm development, and often involve an extended duration of antibiotics and device removal [6].

A link between psychologic distress, such as in anxiety/depression, and poor wound healing has been established [7]. Increased pro-inflammatory cytokines have been shown in patients with experimentally-induced blisters after hostile marital interactions, with poorer wound healing in those categorized as having more hostile relationship patterns [8]. A large-scale observational study involving thousands of patients undergoing hip replacement, hernia repair, and varicose vein procedures found that patients with moderate anxiety or depression had an increased probability of wound complications [9]. The same study also showed an association between anxiety or depression with readmission rates and duration of stay. In an examination of psychological variables and early surgical recovery, psychological distress was found to be associated with impaired wound healing and postoperative recovery [10]. Interestingly, an empathic and patient-centered approach to anxious surgical patients has been shown to improve postoperative recovery and wound healing [11].

There is a paucity of data directly examining the relationship between depression or anxiety in the outcomes following penile prosthesis surgery. The object of this study is to assess for correlation between a preoperative diagnosis of depression or anxiety and complication rates after inflatable penile prosthesis (IPP) surgery, with the goal of developing quality improvement studies and initiatives.

## Methods

After obtaining institutional review board approval and consent from patients, our prospectively collected database of prosthetic urology cases was retrospectively reviewed for cases performed from 2020–2022 in the Wake Forest Baptist Health system, numbering 284 surgeries. 5 Patients who received malleable implants were excluded from the study as they are associated with lower complication rates, for a total of 279 cases, which was calculated to be sufficient for a power of over 0.8 with respect to the statistical analyses performed in this study. All cases were performed in a standardized fashion by a single surgeon using a penoscrotal approach and antibiotic coverage with vancomycin and gentamicin, Procedures were classified as either primary placement of prosthesis or secondary surgery, which included removal with replacement and revision. Revision was defined as any operation including exchange of components, while replacement was defined as replacing the entire device. Complications were defined as any deviation from the expected postoperative course and graded by the Clavien-Dindo classification [12] and identified based on documentation by urologists or emergency room physicians. Times to complication, infection, and reoperation were defined as number of days from date of surgery. Follow-up was based upon last in-person assessment.

Presence of preoperative diagnoses of depression or anxiety was determined by chart review for the related ICD codes or clinical documentation by a provider prior to the surgery. Patients were further classified as having received treatment for anxiety/depression if they reported taking a selective serotonin reuptake inhibitor (SSRI), serotonin norepinephrine reuptake inhibitor (SNRI), atypical antidepressant, or tricyclic antidepressant at the time of their procedure. As contemporary management of depression and anxiety follows similar clinical algorithms, the presence of associated medications was not used to define presence of either condition. Preoperative presence of depression or anxiety, as well as treatment for these conditions, was determined separately for each procedure if patients had multiple operations.

Descriptive statistics for continuous and categorical values were calculated. Mann Whitney U test was used to compare continuous variables as they were found not to be normally distributed by Shapiro-Wilk test. Fisher’s exact test was used to compare the distribution of complications and reoperation based on preoperative mental health status and whether patients received medical management of mental health. Similar analyses were repeated for primary and secondary surgeries. Survival analysis was conducted using Kaplan Meier curves and compared using the Log-rank (Mantel-Cox) test. Patients were censored at time of complication or at last follow up if no complication occurred. Multivariable logistic regression was used to analyze the impact of having a history of treatment or diagnosis of anxiety/depression upon incidence of postoperative complication, controlling for confounding factors such as age, body mass index (BMI), diabetes, smoking status, whether the surgery was primary, prior pelvic surgery, Peyronie’s disease, pelvic radiation, and whether antiplatelet agents were held for 10 days prior to procedure. All of these variables were defined by clinical documentation. Patients missing any of the data points were excluded from the model for that particular data point. Hazard ratios and odds ratios are reported with 95% confidence intervals (CI) and p-values of 0.05 were used for all analyses.

## Results

279 IPP cases were identified, after the 5 malleable prosthesis surgeries were excluded from the 284 eligible cases. 191 (68.46%) surgeries were in white patients, 76 (27.24%) in African American patients, and 12 (4.30%) in patients of unknown/other race. 220 (78.85%) surgeries were primary procedures, and 59 (21.15%) surgeries were replacement/revision procedures. The mean age at time of surgery was 64.25 ± 9.25 years old (37–86 years old) and mean BMI was 30.24 ± 5.11 kg/m^2^ (18.36–46.17 kg/m^2^), with one patient having unknown BMI. 31 (11.11%) patients were smokers or had quit within 6 weeks of surgery. 115 (41.22%) patients had diabetes at time of surgery. 83.87% of patients held blood thinners prior to surgery. Median follow up was 255 days (IQR 127–555 days). 26/279 (9.32%) of all operations had complications, with 7 Clavien 1 complications, 5 Clavien 2 complications, and 14 Clavien 3b complications. 10 cases had some type of postoperative infection (3.58%), and 14 (5.02%) required re-operation/revision. Non-infectious complications included 4 reservoir malfunctions, 1 pump malfunction, 1 cylinder malfunction, 1 unknown malfunction, 1 hematoma, 2 cylinder herniations, 1 reservoir erosion, and 5 miscellaneous complaints including inflammation, pain, and/or poor wound healing. The rate of complication in primary surgery was 7.27% (16/220), while the rate of complication in revision or replacement surgery was significantly higher at 16.95% (10/59) (*p* = 0.040).

Of the 10 cases of postoperative infection, none of these occurred within 30 days of surgery. 4/10 (40%) were superficial wound concerns managed with antibiotics and no need for additional surgery. Of the 6 cases that had subsequent surgery for infectious concerns, 3/6 were able to retain a device based on operative findings. Of the 3 cases that required full device removal, one involved a combination procedure with IPP and artificial urinary sphincter placement, and the rest (2/3) consisted of primary IPP placements with one removed between 2 and 3 months postoperatively, and the other at over 90 days after surgery. Thus, 1/220 (0.45%) primary implant placements required removal for infection within 90 days.

74 patients (26.52%) had either a diagnosis of depression or anxiety prior to IPP surgery, with 51 (18.28%) patients diagnosed with anxiety and 69 (24.73%) patients diagnosed with depression. 56 (25.45%) of patients undergoing primary surgery and 18 (30.51%) of patients undergoing replacement/revision surgery had either depression or anxiety. Mean age was significantly younger among patients with depression or anxiety (62.24 ± 9.25 vs 64.98 ± 9.16 years; *p* = 0.023). Mean BMI was higher among patients with depression or anxiety (31.11 ± 5.43 vs 29.93 ± 4.96 kg/m^2^; *p* = 0.182), though not statistically significant. There was no association between depression or anxiety and a diagnosis of diabetes or tobacco history (see Table 1).

**Table 1:** Demographic characteristics of patient population at time of penile prosthesis surgery.

Characteristic	Total	Depression/Anxiety		P value
		Yes	No	
Prosthesis surgery	279	74 (26.52%)	205 (73.48%)	
Primary surgery	220 (78.85%)	56 (20.07%)	164 (58.78%)	
Removal/replacement	52 (18.64%)	16 (5.73%)	36 (12.90%)	
Revision	7 (2.51%)	2 (0.72%)	5 (1.79%)	
Mean Age (years)		62.24 ± 9.25	64.98 ± 9.16	0.023^a^
Mean Body Mass Index (kg/m^2^)		31.11 ± 5.43	29.93 ± 4.96	0.182^a^
Current Smoker				0.669^b^
Yes	31 (11.11%)	9 (3.23%)	22 (7.89%)	
No	245 (87.81%)	63 (22.58%)	182 (65.23%)	
Unknown	3 (1.08%)	2 (0.72%)	1 (0.36%)	
Diabetes				0.495^b^
Yes	115 (41.22%)	33 (11.83%)	82 (29.39%)	
No	164 (58.78%)	41 (14.70%)	123 (44.09%)	
Complications	26			
Infection	10 (38.46%)	6 (23.08%)	4 (15.38%)	
Pain/inflammation/poor healing	8 (30.77%)	5 (19.23%)	3 (11.54%)	
Malfunction	7 (26.92%)	3 (11.54%)	4 (15.38%)	
Hematoma	1 (3.85%)	0	1 (3.85%)	
Clavien Dindo				
1	7 (26.92%)	5 (19.23%)	2 (7.69%)	
2	5 (19.23%)	2 (7.69%)	3 (11.54%)	
3b	14 (53.85%)	7 (26.92%)	7 (26.92%)	

^a^These values were calculated with a Mann-Whitney test.

^b^These values were calculated with Fischer’s exact test.

Presence of either mental health condition (depression or anxiety) was associated with a significantly higher likelihood of postoperative complication (OR 3.753; 95% CI: 1.671–8.822; *p* = 0.002). Depression (OR = 4.200; 95% CI: 1.859–9.934; *p* = 0.001) and anxiety (OR 3.905; 95% CI: 1.735–8.970; *p* = 0.002) were also separately associated with the higher rate of overall complications. There was no association between depression and complications for primary surgery (OR 2.828; 95% CI: 1.069–7.679; *p* = 0.061), but a significant association was found for complications after replacement or revision (OR = 8.061; 95% CI: 1.628–31.03; *p* = 0.006). Like depression, anxiety was not significantly associated with complication for primary surgery (OR 3.000; 95% CI: 0.9996–8.225; *p* = 0.083) but was for replacement/revision surgery (OR 7.167; 95% CI: 1.526–30.08; *p* = 0.014). Patients with depression or anxiety had a higher infection rate at 8.11% compared to 1.95% in patients without either diagnosis, corresponding to a OR of 4.434 (95% CI: 1.139–14.17; *p* = 0.024). Both depression (OR 4.929; 95% CI: 1.262–15.76; *p* = 0.017) and anxiety (OR 4.848;, 95% CI: 1.493–15.53; *p* = 0.021) were separately found to be associated with increased rates of postoperative infection.

**Fig. 1:**
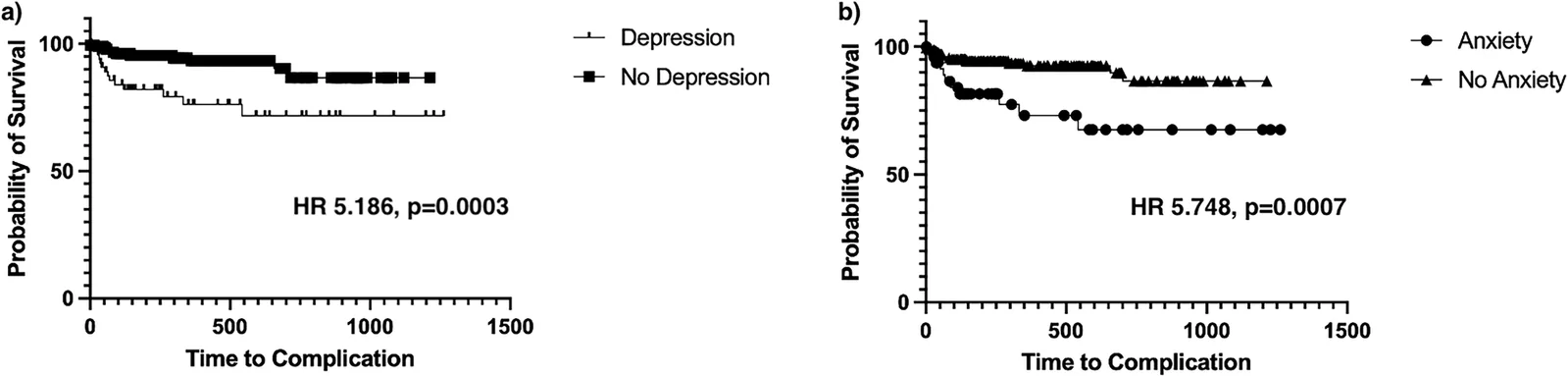
Time to complication for patients with depression or anxiety. **a** Survivorship free of complication for patients with depression versus those without. **b** Survivorship free of complication for patients with anxiety versus those without.

Survival analysis found that patients with either depression or anxiety had a significantly increased risk of complication and a shorter time to complication. A diagnosis of depression was associated with a hazard ratio (HR) of 5.186 for having a complication (95% CI: 2.105–12.78; *p* = 0.0003; Fig. 1a), and an average time to complication of 121.2 days versus 208.8 days for those without depression. A diagnosis of anxiety was associated with a hazard ratio of 5.748 for complication (95% CI: 2.079–15.89; *p* = 0.0007; Fig. 1b), and an average time to complication of 147 days versus 172.4 days for those with no anxiety. A multivariable logistic regression model was used to assess the association of multiple confounding factors with IPP complications previously reported in other studies (Table 2), showing that having a diagnosis of anxiety or depression and having a primary surgery were the independent predictors of complication with an OR of 3.629 (95% CI: 1.426–9.478, *p* = 0.007) and 0.2938 (95% CI: 0.115–0.758, *p* = 0.012) respectively.

**Table 2:** Multivariable logistic regression analysis of risk factors for complication after penile prosthesis surgery.

Variable	Estimate	Standard Error	Odds Ratio	95% Confidence Interval of Odds Ratio	P-value
Age at surgery	−0.04137	0.02549	0.9595	0.9120–1.009	0.1051
Body mass index	−0.04413	0.04929	0.9568	0.8655–1.051	0.3640
Current smoker	−0.7651	0.8209	0.4653	0.06657–1.920	0.3168
History of radiation	−0.4799	0.8259	0.6188	0.08802–2.602	0.5438
History of radical prostatectomy	0.2467	0.5156	1.280	0.4492–3.454	0.6347
History of inguinal hernia repair	0.7545	0.5289	2.127	0.7250–5.907	0.1638
Diabetes	−0.6060	0.5080	0.5455	0.1914–1.433	0.2230
Depression/Anxiety	1.289	0.4784	3.629	1.426–9.478	0.0070
History of Peyronie’s disease	−0.8853	0.6449	0.4126	0.1019–1.339	0.1468
Primary surgery	−1.225	0.4769	0.2938	0.1146–0.7579	0.0119
Blood thinners held for 10 days pre-operatively	−0.7541	0.5745	0.4704	0.1575–1.548	0.2042

Conversely, patients without evidence of these mental health conditions or those who had filled a prescription for active treatment prior to surgery had no significant change in risk of complication compared to those with untreated anxiety/depression (OR 0.349; 95% CI: 0.093–1.242; *p* = 0.131). There was no significant relationship between medical treatment of depression or anxiety preoperatively and subsequent complication when only patients with those diagnoses were analyzed (OR 0.823; 95% CI: 0.211–3.133; *p* = 0.721). Survival analysis found no significant difference in time to complication in patients treated for depression or anxiety versus those that were not (HR 0.348; 95% CI: 0.067–1.812; *p* = 0.210).

The subset of patients with anxiety had a significantly increased risk of reoperation (OR 3.667; 95% CI: 1.181–11.50; *p* = 0.026). This increased risk in anxious patients was seen for primary surgeries (OR 4.889; 95% CI: 1.358–17.25; *p* = 0.038), but not for replacement or revision surgeries (OR 2.444; 95% CI: 0.408–12.34; *p* = 0.310). When examining the subset of patients with depression, there was a narrowly significant increase in risk of reoperation (OR 3.274; 95% CI: 1.208–8.772; *p* = 0.049). Interestingly, no significant association between depression and reoperation was found after primary surgery (OR 3.511; 95% CI: 0.984–12.35; *p* = 0.086) or removal/replacement (OR 2.533; 95% CI: 0.534–11.61; *p* = 0.357). Similarly, patients who had their anxiety or depression managed by medication did not have a significant difference in reoperation compared to those managed without prescription medication (OR 0.671; 95% CI: 0.101–7.660; *p* = 0.522).

**Fig. 2:**
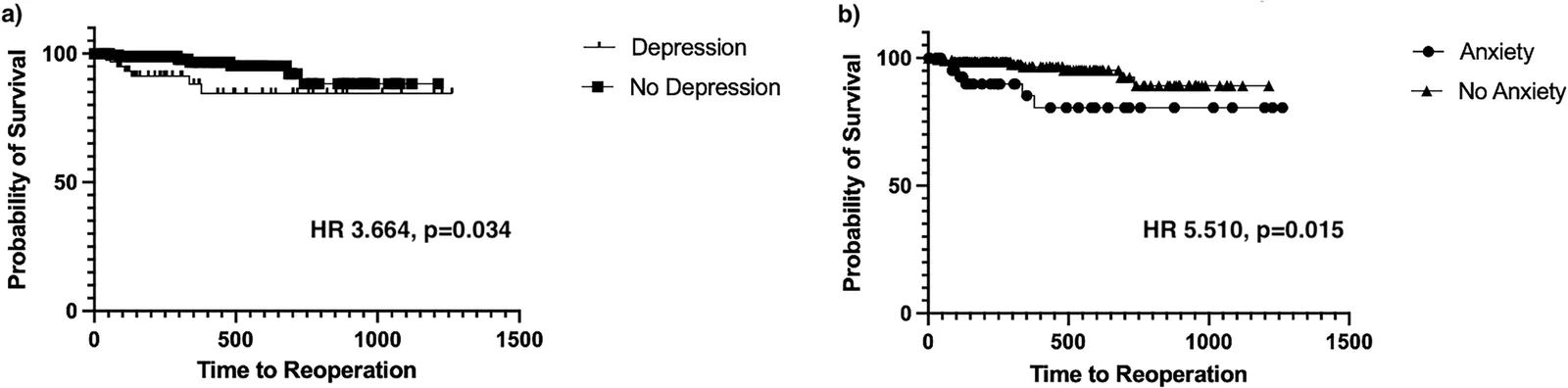
Time to reoperation for patients with depression or anxiety. **a** Survivorship free of reoperation for patients with depression versus those without. **b** Survivorship free of reoperation for patients with anxiety versus those without.

Survival analysis showed that both patients with anxiety and patients with depression had shorter times to reoperation than patients without either diagnosis. Depression was associated with a HR of 3.664 for re-operation (95% CI: 1.098–12.22; *p* = 0.034), with an average time to reoperation of 160 days compared to 383 days for those without (Fig. 2a). Anxiety was associated with a HR of 5.510 for reoperation (95% CI: 1.402–21.65; *p* = 0.015), with an average time to reoperation of 181.2 days compared to 339.25 days for those without (Fig. 2b). Patients medically treated for depression or anxiety did not have a significant difference in time to reoperation than patients that did not receive treatment (HR 0.986; 95% CI: 0.1265–7.631; *p* = 0.989).

## Discussion

Depression and anxiety are commonly comorbid with erectile dysfunction (ED), and the onset of ED often precipitates poor mental health [13]. Both depression and anxiety have been linked to poor surgical outcomes, with a large review finding increased wound complications in patients with moderate depression or anxiety across four of the most common surgical procedures performed in England [9]. Though postoperative complications present a major source of morbidity in IPP surgery and often lead to reoperation [14], the association between preoperative anxiety and depression and outcomes after IPP placement/revision has not been explored in the literature until now. We found that over a quarter of men undergoing these operations had depression or anxiety, and that these specific patients had a significantly greater rate of surgical complications.

The prevalence of depression among men with ED is high, and the relationship is thought to be bi-directional [15]. The Massachusetts Male Aging Study established that 12% of patients that developed ED had pre-existing depression [16]. Similarly, a large Finnish study found that 7% of men with new onset ED had depression [17]. ED appeared to have an even stronger association with developing depression, with rates of onset as high as 46% in a cohort of young males [18]. Our study was consistent with this data as 25% of our patients had a diagnosis of depression, though the temporal relationship of depression and ED is unknown.

The data regarding the relationship between anxiety and ED is less robust, as generalized anxiety disorder is often coupled with depression in analysis. Jern et al. and Manalo et al. both identified that symptoms of anxiety and depression were associated with poor erectile function [13, 19]. A cross-sectional survey of Chinese ED patients found 79.82% suffered from anxiety [20]. A retrospective study of ED patients in India found that 23.4% had some kind of anxiety disorder, mostly predating ED [21]. Our data showed that 18.3% of our patients had anxiety, though our cohort was clearly different with respect to age and nationality. Notably, panic disorder and post-traumatic stress disorder (PTSD) are also linked to ED, with studies showing that panic disorder confers around a 33% increased risk of ED while PTSD confers an almost 4-fold increased risk [22, 23].

The connection between depressive and anxious symptoms and poor wound healing is hypothesized to be secondary to changes in cytokines, with an early study by Kiecolt-Glaser et al. showing reduced IL-1beta, an interleukin involved in tissue remodeling, in patients exposed to stress [24]. Depressive symptoms are also correlated with increases in plasma IL-6 and TNF-alpha, which promote both local and systemic inflammation via the hypothalamus-pituitary-adrenal axis as part of the acute phase response [25, 26]. The modulation of these interleukins differs however, as glucocorticoids suppress the release of TNF alpha but not IL-6 during prolonged stress [27]. Prolonged stress also induces a counterregulatory response in leukocytes to become more resistant to glucocorticoid stimulation [28], which may lead to overall systemic inflammation but reduced local function of immune cells in wounded areas. Jian et al. found that TNF-alpha, IL-6, and IL-1beta were all increased in rats exposed to aversive stimuli before and after the creation of a wound, which correlated with poorer wound healing rates [29]. In humans, hostile marital couples were shown to have higher baseline levels of IL-6 and TNF alpha after a conflict, but had lower local cytokine production at the site of an induced blister as well as slower healing of the wound [30]. Higher stress scores were also correlated with higher cortisol, as well as lower levels of both IL-1alpha and IL-8, two key proinflammatory cytokines in early wound healing, at induced wound sites in a separate study [31]. Furthermore, there is evidence for a bidirectional relationship between depression and inflammation measured by C-reactive protein in a 6-year frame [32].

The overall complication rate of primary IPP surgery has been reported as around 5% [14], but may be as high as 25% for revision cases [33]. However, these rates may not account for device malfunction, which may occur in up to 17.5% over 10 years [4]. Infection is often the most feared complication given the risk of device loss, and has been reported after 1–3% of cases in the era of antibiotic coatings and washouts [2]. Our data in this relatively short window of collection is similar to existing data, with a 7.2% and 16.1% complication rate for primary and revision/replacement cases, respectively. The overall infection rate was 3.5%, but only 0.5% of primary implantation surgery infections were severe enough to require removal. Malfunction was seen in only 2.5% and may reflect the length of follow-up. With respect to reoperation rates, estimates range from 11–15% over 5–10 years [34]. While our reoperation rate of 4.9% may appear low due to the length of follow-up, it is lower than that reported in studies with similar follow-up [35, 36].

We found a significant association between depression and anxiety with postoperative infection. This mirrors similar findings among other types of prosthetic surgery. In hip and knee replacements, depression and anxiety have been linked to increased risk of both superficial and prosthetic infections in primary and revision procedures [37, 38]. Depression was also associated with infection after ventricular assist device implantation, which was attributed to poor compliance with device care [39]. In addition to the hypothesized inflammatory derangements increasing the risk of poor wound healing and infection, poor device care may also contribute to the increased risk of IPP infection in the setting of depression or anxiety. Notably comorbidities of depression/anxiety, such as substance use and diabetes, may be considered additional risk factors for device infection [40–42]. Management of mental health may reduce the risk of infectious complications, as a randomized control trial measuring the effect of cognitive behavioral therapy on depressed heart bypass patients showed a reduction in postoperative infectious illnesses [43].

Our results also suggest a relationship between depression and anxiety and the need for reoperation. Of note, a similar relationship between mental health and reoperation has been established for orthopedic prosthesis as well [37]. Infection and device malfunction are the most common reasons for penile prosthesis revision [34] and also account for the majority of complications in this study. Though the association between psychological factors and infection have been discussed, it is unclear if there is any association between mental health and reports of device malfunction. Depression and anxiety prior to surgery are associated with worse pain control and increased analgesic requirements [44], which may make patients more sensitive to a malfunctioning prosthesis. Depression in elderly patients is also a risk factor for progression to mild cognitive impairment [45]. As most penile prosthesis recipients are elderly, depression-related cognitive impairment could contribute to reported malfunction rates as a function of device misuse.

The findings in this study suggest preoperative depression and anxiety negatively affect long-term outcomes of IPP surgery. The postoperative complication and infection rate in patients with anxiety or depression are comparable to those reported for risk factors such as spinal cord injury and diabetes [46, 47]. The current consensus in preventing complications in the preoperative setting is to screen for relevant risk factors and to optimize their management, such as employing A1c cutoffs in diabetic patients [48]. Identification and management of depression and anxiety could potentially improve outcomes in nearly a quarter of IPP cases, as studies suggest optimizing mental health improves recovery [11]. Given that depression is associated with increased length of stay, readmission rates, and overall cost of care, preoperative efforts may reduce the burden on health care systems [49].

This study has multiple limitations, some of which have already been highlighted. It is retrospective from a single referral-based tertiary care center with a relatively small sample size, and thus results may not be fully generalizable to other practice settings. Patients tended to be mostly white and socioeconomic/educational background was not assessed, which could lead to bias when generalizing these results to the population. Confounding variables such as adherence to wound care, nutrition, and use of illicit substances were not routinely documented in the chart postoperatively and thus not included in analysis. There was also relatively little psychiatric history that could be gleaned through chart review outside of diagnosis and medication usage. No data regarding severity of psychiatric illness based on standardized instruments or information regarding prior psychotherapy was available on chart review. As depression and anxiety are best managed through a combination of medication and therapy, we were unable to fully assess whether patients were adequately managed preoperatively, and we lack a definition of what fully ‘optimized’ would entail. Finally, though we assessed patient charts for ICD codes consistent with depression or anxiety, there was no way to determine whether these codes accounted for past episodes of psychiatric illness or currently active disease. These factors may account for the lack of association between recorded treatment of mental health conditions and our outcomes.

## Conclusion

In summary, diagnoses of depression and/or anxiety are common among men undergoing IPP surgery, and are associated with increased complications, infections, and reoperation. Though this association has been reported by other surgical subspecialties, this study is, to our knowledge, the first to establish such a relationship in IPP recipients. Appropriate pre-operative mental health screening and management of depression and anxiety represent an opportunity for urologists to potentially improve patient outcomes. Further study will be needed to determine optimal assessments and therapeutic targets prior to surgery.

## Data Availability

Data generated for this study can be found within the published article. Additional data is available from the corresponding author upon request.
